# Exercise in regional breast cancer with neoadjuvant anthracycline-based chemotherapy with immune checkpoint-inhibition: study protocol for a prospective randomised controlled trial

**DOI:** 10.3389/fonc.2026.1742136

**Published:** 2026-06-05

**Authors:** Elena Murr, Sabine Grill, Marion Kiechle, Anne Kraemer, Bernadine Madl, Simon Glowka, Bernhard Haller, Caterina Fiorentini, Emmanuel Adjej, Mark J Haykowsky, Martin Halle, Simon Wernhart

**Affiliations:** 1 Department for Preventive Sports Medicine and Sports Cardiology, TUM School of Medicine and Health, Technical University Munich (TUM), Klinikum rechts der Isar, Munich, Germany; 2 Department of Gynecology and Obstetrics, TUM School of Medicine and Health, Technical University Munich (TUM), Klinikum rechts der Isar, Munich, Germany; 3 Institute of Artificial Intelligence (AI) and Informatics in Medicine, TUM School of Medicine and Health, Technical University Munich (TUM), Klinikum rechts der Isar, Munich, Germany; 4 Integrated Cardiovascular and Exercise Physiology and Rehabilitation (iCARE) Laboratory, College of Health Sciences, University of Alberta, Edmonton, AB, Canada; 5 Department of Cardiology, Hochgebirgsklinik, Davos, Switzerland; 6 Deutsches Zentrum für Herzkreislaufforschung (DZHK) (German Centre for Cardiovascular Research), Partner Site Munich Heart Alliance, Munich, Germany

**Keywords:** chemotherapy, exercise capacity, exercise training, immunotherapy, triple negative breast cancer

## Abstract

**Background:**

Anthracycline-based chemotherapy and immune checkpoint inhibition-increasingly used together in triple negative breast cancer (BC)-both cause cardiotoxicity and decline in peak oxygen consumption (VO2peak). While guidelines recommend exercise training for BC, the effectiveness of endurance, strength, and balance training during neoadjuvant immunochemotherapy remains unclear.

**Methods:**

The ExACT-ICI (Exercise in Regional Breast Cancer with adjuvant and neoadjuvant Anthracycline-based ChemoTherapy with Immune Checkpoint-Inhibition, NCT06672120) trial is a prospective single-center study randomizing regional TNBC patients (stage I-III, ages 18-65) receiving anthracycline chemotherapy plus pembrolizumab into exercise training (ET) or usual care groups. ET participants complete five weekly video-supervised sessions (endurance, resistance, balance) during 24-week treatment, monitored via smartwatch. Both groups undergo cardiopulmonary exercise testing, stress echocardiography, and strength/balance assessments at baseline, 6, 12, and 24 weeks. The primary outcome is VO2peak change from baseline to 24 weeks, with secondary outcomes including physical function, cardiac biomarkers, echocardiographic variables, quality of life, strength, balance, and exploratory cardiac adverse events through 52 weeks.

**Discussion and limitations:**

This study is the first to assess the effects of exercise intervention on VO2peak during immunochemotherapy in TNBC patients. Repetitive testing and smart watch monitoring will ensure optimized exercise prescription and safety during supervised, video-based training sessions. The study is limited to patients able to participate in online training sessions with a digital device.

## Graphical Abstract

**Figure:**
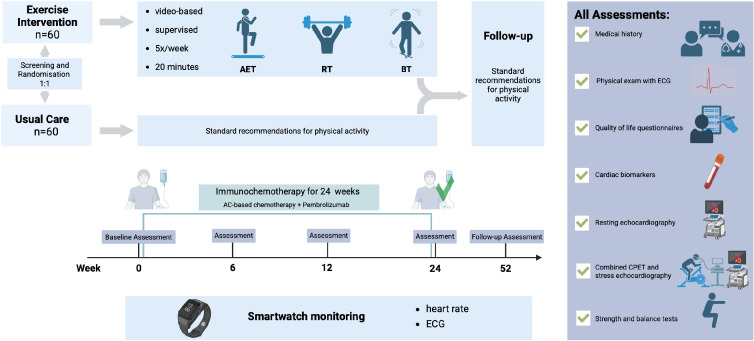
Patients are randomized 1:1 into an exercise intervention and usual care group. The intervention group receives 24 weeks of combined aerobic endurance (AET), resistance (RT) and balance training, BT (5x/week, 20 minutes each). Training is video-based and supervised. Patients in usual care receive standard recommendations on physical activity. Assessment in both groups is performed at baseline before initiation of immunochemotherapy (anthracycline, AC-, based chemotherapy combined with Pembrolizumab), after six, 12 and 24 weeks. Assessment includes a medical history, bioimpedance measurement, physical examination, questionnaires on quality of life, cardiac biomarkers, electrocardiography (ECG) and resting echocardiography. Exercise capacity is assessed with combined cardiopulmonary exercise testing (CPET) and stress echocardiography. Strength and balance test are performed during every visit. After 24 weeks both groups receive standard recommendations for exercise and are followed- up after 52 weeks. Heart rate is monitored with smart watches throughout the study period and symptom- triggered ECG is documented. Images created with biorender.com.

## 1 Introduction

Breast cancer is the most commonly diagnosed cancer in women worldwide (1), with triple negative breast cancer (TNBC) comprising 10-15% of cases (2). Anthracycline-based (AC) chemotherapy is an established treatment concept for TNBC and is increasingly combined with immune checkpoint inhibitors (ICI), such as pembrolizumab (inhibition of programmed cell death protein 1/PD1) (2). These treatments can cause cancer therapy-related cardiac dysfunction (CTRCD), characterized by reduced left ventricular ejection fraction, decreased global longitudinal strain, and elevated cardiac biomarkers (3). Moreover, peak oxygen uptake (VO2peak) can decrease up to 25% following neoadjuvant chemotherapy (3) as a consequence of both central (cardiac output) and peripheral (oxygen extraction) abnormalities (4).

Exercise training is an effective intervention to mitigate treatment-related reductions in VO2peak (8-10); however, the mechanisms responsible for this improvement remain unclear. Several studies, in the (neo)adjuvant setting reported an increase in VO2peak during AC-based chemotherapy, while no improvement of resting systolic and diastolic function has been observed (5). In contrast, the recent BREXIT trial found that one-year of exercise training increased peak exercise VO2 and cardiac output in BC survivors treated with AC- chemotherapy (6). Despite promising results with AC-based chemotherapy, the effectiveness of exercise intervention during combined immunochemotherapy for TNBC remains unknown. Moreover, the incidence of CTRCD, heart failure, atrial fibrillation, and ICI-myocarditis in this population is also unclear.

The Exercise in regional breast cancer with neoadjuvant Anthracycline-based ChemoTherapy with Immune Checkpoint-Inhibition (ExACT-ICI, NCT06672120) trial will address this knowledge gap by analyzing VO2peak and its central and peripheral components along the oxygen cascade during exercise using combined cardiopulmonary exercise testing (CPET) and stress echocardiography. This comprehensive approach will help tailor exercise prescriptions to individual patient needs and will inform future cardio-oncological exercise guidelines, while the digitally supervised format will offer a cost-effective treatment option for the growing breast cancer population.

## 2 Methods and analysis

### 2.1 Study objectives

The objective of the study is to evaluate the effect of a video-based, supervised, combined ET (aerobic endurance training, AET, resistance training, RT, and balance training, BT) on VO2peak (primary outcome), rest and exercise cardiac function (stroke volume, cardiac output, and estimated arterial-venous oxygen difference), strength, balance, physical function, and quality of life (secondary outcomes) in TNBC patients. We hypothesize that the change in VO2peak from baseline until the end of immunochemotherapy will be superior in ET compared to UC. We expect ET to improve VO2peak during ET, while UC is expected to decrease. We also state that ET will be superior to UC in improving quality of life, upper and lower body strength, cardiac output and arteriovenous oxygen difference.

### 2.2 Trial design

The ExACT-ICI-Trial is a prospective two-armed, 1:1 (n=60/group) randomised, monocentric, intervention trial and is currently starting to recruit, the first patient inclusion was on 03-04-2025. Recruitment is expected to continue until December 2027. The entire trial is expected to last for 2 years and 3 months (for a timeline see **Supplementary Figure 1**, for enrolment see **Supplementary Figure 2**). For patient referral there is a collaboration with urban Breast Cancer Centers and clinics of Gynecology and Obstetrics. Patients undergo a total observation period of 52 weeks, starting with the intervention phase (baseline assessment, t0, until the end of combined immunochemotherapy after 24 weeks, t24), followed by a follow-up phase until week 52 (t24-t52). Involved trial physicians recruit eligible patients attending their gynecological pre-treatment consultation. Baseline assessment will be performed within a week after initial presentation. Each inclusion interview is documented according to the CONSORT scheme (7), regardless of whether the patient ultimately agrees to participate, or not. If patients drop out of the study due to health complications, the exclusion is documented with reasons according to the CONSORT scheme, the same applies if a patient successfully completes the study. Study physicians are blinded to the intervention status of the patients from the beginning. Final unblinding is carried out when all data sets are available at the end of the study. In case of serious adverse events, such as ventricular arrhythmia or ICI-associated myocarditis, the attending physician will be unblinded to maintain patients’ safety. Further medical proceedings are discussed in an interdisciplinary context and patients are excluded from the study. In case of dose reductions or changes of treatment, exercise training will continue until the end of immunochemotherapy to represent a real-world setting. Since high-volume centers are involved, we do not expect that potential individual treatment prolongation will affect the overall schedule of the study.

### 2.3 Randomization

The allocation sequence is computer-generated and was determined in advance. The local Institute of AI and Informatics in Medicine provided sequentially sealed envelopes, labelled and numbered from ExACT001 to ExACT120. Upon recruitment, the corresponding envelope is opened and documented by the attending research assistant.

### 2.4 Eligibility criteria

Female patients are eligible for study inclusion with newly diagnosed, local TNBC (stage I-III) ≥ 18 to 65 years of age scheduled for immunochemotherapy with pembrolizumab and anthracycline-based chemotherapy. Eligibility is assessed in the interdisciplinary tumour board for breast cancer screening patients. Per definition, TNBC has low expression of oestrogen receptor, progesterone receptor and HER2 (8). BC patients with an oestrogen/progesterone receptor expression of up to 20%, HER2 expression of 1+ or 2+ and FISH-negative status can be recruited as well as they are also eligible to this therapy regimen. Even though there are rare cases of male TNBC, for the comparability of research data, only females are included. Exclusion criteria are unstable cardiac conditions (clinical suspicion of progress or unstable coronary artery disease, signs of acute heart failure, haemodynamically relevant arrhythmias) or orthopaedic disability to exercise. It is a prerequisite to maintain safety during the supervised online training. Thus, a major language barrier or cognitive inability to follow instructions during exercise is an exclusion criterion because patients must be able to understand and follow training instruction. Patients must be willing to attend the online training sessions with a digital device and should not have a major mobility impairment that would fundamentally limit the participation in ET. Informed consent must be signed to attend the online training sessions.

### 2.5 Study outcomes

The primary outcome of the study is the difference of VO2peak between baseline to the end of intervention per group (t0, baseline, to t24, end of neoadjuvant immunochemotherapy) during cardiopulmonary exercise testing (CPET). We hypothesize that the intervention group is superior to the control group and suspect a minimum difference of 2.0 mL/kg/min, which is considered clinically relevant (6). CPET will be performed using upright bicycle ergometry (Lode™ Excalibur Sport) and breath-by-breath analysis (Cortex Metalyzer^®^ 3B). Exertional exercise testing (defined by reaching a respiratory exchange ratio of >1.05 and a BORG scale >17 from 20 points) will be performed using a ramp protocol and aiming at an exercise duration of 8–12 minutes. VO2peak will be determined averaging the last 30 seconds of exercise testing. Secondary outcomes include the difference of VO2peak between t0 to the end of follow-up (t52) per group as well as the difference of VO2peak between t24 and t52 per group. Burden of atrial fibrillation (AF), performance in short physical performance battery (SPPB), improvement of muscle strength of the upper (biceps curl) and lower extremity (knee extensor), changes of resting and stress echocardiographic variables (GLS, LVEF, and diastolic function, tricuspid annular plane systolic excursion, TAPSE, estimated arteriovenous oxygen difference), cardiac biomarkers, change in EORTC QLQ-C30 (European Organization for Research and Treatment of Cancer Quality of Life Questionnaire) as a marker for quality of life, decrease of VE/VCO2 during CPET, the incidence of CTRCD and ICI-myocarditis will be assessed from t0 to t24, t0 to t52 and t24 to t52.

### 2.6 Assessment and intervention

Both groups will receive the same treatment regimen for TNBC: 12 weeks of weekly paclitaxel combined with carboplatin and pembrolizumab, followed by 12 weeks of epirubicin combined with cyclophosphamide and pembrolizumab every 3 weeks. Every patient receives five examinations to monitor cardiopulmonary exercise capacity: One baseline assessment (t0) before the beginning of immunochemotherapy, then after 6, 12 and 24 weeks as well as one follow-up assessment after 52 weeks (Graphical Abstract). Patients are instructed not to exercise the day before the respective examinations to avoid exercise-induced troponin elevation independent of cardiac disease.

#### 2.6.1 Assessment

The workflow of assessment visits is shown in **Table 1**. The examinations start with a medical history, a standardised physical examination and a 12-lead electrocardiogram (ECG). A resting echocardiography is performed following guideline recommendations (9), CPET is performed according to current guidelines (10). Blood samples are taken to determine troponin I and NTproBNB levels. CPET is performed on a tilted table with a ramp protocol aiming at an overall exercise duration between 8–12 minutes with simultaneous bicycle stress echocardiography. Standard CPET variables are determined as well as stroke volume reserve, heart rate development, estimated arterial-venous oxygen difference, estimated systolic pulmonary pressure (sPAP), TAPSE and diastolic function at peak exercise (11, 12). Body composition is assessed with bioimpedance measurement. Estimated arterial-venous oxygen difference will be calculated as VO2/cardiac output during exercise.

**Table 1:** Workflow of assessment.

Exams	t0	t6	t12	t24	t52
Verification of in- and exclusion criteria	x				
Randomization	x				
Medical history, physical exam, anthropometry, blood samples*, ECG, resting echocardiography, bioimpedance	x	x	x	x	x
Cardiopulmonary exercise testing and stress echocardiography	x	x	x	x	x
Strength testing (upper body, legs)	x	x	x	x	x
Testing of physical functioning (SPPB)	x	x	x	x	x
Questionnaires	x	x	x	x	x
Smart watch monitoring	x	x	x	x	x
Documentation of adverse events	x	x	x	x	x

ECG, Electrocardiograph; SPPB, Short physical performance battery, testing of physical functioning. * Patients are asked to keep 48 hours of physical rest before cardiac biomarkers are taken. t0-t52: Visits at baseline (t0), and after weeks six (t6), 12 (t12), 24 (t24), and 52 (t52). All patients receive telemetric monitoring throughout the study and are educated to carry the smart watch for most hours of the day during the study.

Functional status is assessed with the short physical performance battery, SPPB (achievable points from 0-12, 12 being the best functional status) (13), Karnofsky-Index (assessment of the subjective performance status, from 10 points, moribund, to 100 points, normal unrestricted activity as pre diagnosis) (14). Balance is assessed with the MCTSIB (modified clinical test of sensory interaction in balance) (15), measuring the commuting speed by using a force plate, and strength is tested using three different exercises: Grip strength (three attempts with the dominant hand, quantified by using a digital hand dynamometer resulting in maximum force in kg), maximum time of plank and wall sit (seconds of achieved activity). Quality of life is assessed with the Kansas City Cardiomyopathy Questionnaire (16) and the EORTC QLQ-C30 of the European Organization for Research and Treatment of Cancer (17).

If a patient is randomised to ET, training heart rates are prescribed for AET in the supervised, video-based training sessions between the first and second ventilatory threshold (increasing in intensity during the intervention period). The level of complexity for RT and BT are categorized (low, medium, high) and adapted to the current needs of the patients. In general, technical accuracy of the performed exercises must be ensured by the coach before intensity can be increased. Patients randomised to UC are advised to adhere to standard principle of physical activity (18, 19).

#### 2.6.2 Smart watch monitoring

Every patient receives a smartwatch for the complete duration of the trial irrespective of group allocation. Patients are advised to trigger an ECG with the smartwatch three times a day (8.a.m., 2.p.m., 8.p.m.) and whenever symptoms such as palpitations, chest pain or dizziness are experienced. At the beginning and end of each training, ECG documentation is also performed. ECG and heart rate data are transferred to a telemedicine-unit, where a physician analyses the results daily. Detection of arrhythmia necessitating therapy leads to direct patient contact by a physician. Patients receive a physician emergency number to report any symptoms during participation in the study.

#### 2.6.3 Exercise training intervention group

The intervention group receives five supervised training sessions per week (Monday to Friday, 20 minutes each) over the entire duration of therapy (24 weeks). Every session contains a warm-up and cool down phase, balance training (BT) is included in all sessions at the beginning to improve muscle-, tendon- and bone interplay and prevent further decline of mineral density (20). We use a non-linear step-up approach to increase exercise intensity over time: AET starts at an intensity level of five heartbeats above the first ventilatory threshold and is increased up to the second ventilatory threshold over time. The coaches are required to verify the heart rate zones after each session (coaches do have access to the heart rate monitoring system of telemedicine). Elements of both moderate continuous and high-intensity interval training are implemented to create variation of training content and to mirror real-life exercise training. This typically implements both moderate continuous and high intensity interval training, which complement each other during training intervention. As higher intensities are physiologically more demanding, this type of intervention may be better feasible at the end of each chemocycle. It will be at the discretion of the sports scientist to individually tailor these modes of training to every patient. Although five sessions per week seem burdensome for the patients, we have received positive feedback on this approach in preliminary exercise sessions during immunochemotherapy in ten patients during the entire duration of immunochemotherapy. The content (percentage of endurance, resistance and balance per session) of the exercise sessions can be varied by the trainer, depending on the patients` well-being: For instance, on the day of chemotherapy, balance training may be emphasized to “mobilize” patients and counteract symptoms of polyneuropathy (“active recovery” rather than omission of an exercise session). This approach has been introduced in clinical practice in our department and is taken on very well by the patients.

Every patient of ET receives training materials (such as dumbbells, heavy hands, elastic bands) required for the workouts. AET focuses on continuous endurance activities such as rope skipping or jumping jacks. Four exercises are performed for three minutes each followed by a one- minute break. RT contains six strength exercises targeting legs (2), back muscles (2), arms/shoulders (1) and core stability (1). Exercises are performed for 45 seconds with a 15- second break. Two sets are performed, the between- sets break is two minutes. RT and BT are increased in complexity over time, provided that technical performance of the exercises are appropriate. RT and BT contain three intensity levels, and the coach reallocates each patient to the most suitable level after every examination.

An information brochure with an overview of the selection of exercises and general training information is handed out to each ET patient at t0 to increase the educational aspect of training. By implementing video-based supervised training and education material we expect a lower drop-out rate and better adherence to ET.

The supervised training sessions are conducted using a video platform, with one trainer coaching a maximum of up to four patients at the same time. Upon patient consent, patients know that they exercise together and interaction during training sessions is possible online, which provides a social element for training and can stimulate motivation. Following the intervention period, general recommendations for exercise according to the current sports cardiology guidelines are provided until the end of follow-up (18, 19). A secured weblink will be made available to all participants to meet in an “online exercise room”, an approach that was approved by the local ethics committee.

#### 2.6.4 Usual care

UC undergoes the same five examinations at t0, t6, t12, t24 and t52 as ET, but only receives guideline recommendations for exercise during the 52-week study period (18, 19).

#### 2.6.5 Criteria for discontinuing or modifying allocated interventions

The close (tele) monitoring of the patients by an experienced cardiologist during every assessment increases the likelihood of early detection of cardiac adverse effects such as ICI- myocarditis or atrial fibrillation (19). If there is clinical suspicion of ICI-associated myocarditis, the patient is hospitalised, and magnetic resonance imaging of the heart is performed. Diagnosis of ICI-myocarditis requiring high-dose steroids leads to study exclusion.

Adverse events are documented by the study personnel and anonymously reported to the study physician who clinically decides whether unblinding needs to be done. Adverse events include hospitalisations, syncope, chest pain, excessive shortness of breath that exceeds the level of exertion, palpitations, or exercise-induced injuries during training. If any of these events occur during testing or supervised training, exercise must be stopped immediately, and the study physician will be informed and decides whether emergency services are necessary. Each event is documented using a patient safety form. Before training is resumed, the study physician needs to clear patients for re-entry into the study. Any clinically relevant changes of health status are immediately discussed between physicians of both gynaecology and sports cardiology departments to ensure patients` safety.

Patients` addresses are known to the trainer. If an adverse event takes place during an exercise session, the training sessions will be stopped immediately, and the trainer (educated in basic-life support, BLS, and detection of cardiac arrest signs) will immediately contact local emergency services and the study physician. Upon study inclusion, relatives of patients are instructed to provide BLS and are encouraged to be present during the patient`s training sessions. In addition to being a potential first responder in case of an adverse event, this can be expected to further increase exercise adherence.

#### 2.6.6 Post-trial care

Patients are encouraged to continue exercise training upon study completion by referring them to local (breast) cancer sports groups; a sports cardio-oncology exam after the study period is recommended.

#### 2.6.7 Sample size calculation

The results of the ACT trial (Optimal Timing of a Tailored Physical Activity Program During Chemotherapeutic Cancer Treatment to Reduce Long-Term Cardiovascular Morbidity), which also used a 24- week exercise intervention for BC patients either during (Group A) or after (Group B) chemotherapy, were used to estimate effects for the primary outcome VO2peak. A standard deviation of VO2peak 2.88 mL/kg/min (Group A) and 2.98 mL/kg/min (Group B) were reported (21). We assumed a standard deviation of 3 mL/kg/min within the groups for sample size calculation. An effect size (Cohen’s d = mean difference between the groups/standard deviation within the groups) of 2/3 (= 0.667) was estimated. Considering these assumptions, 50 patients per group are required to detect a relevant difference between the groups with a power of 90%. Since a drop-out rate of 15% of the participants is expected, n=60 patients will be included into each group, we expect to screen 300 patients to attain our sample size (**Supplementary Figure 2**). The number of cases was calculated using the case number calculation software nQuery Version 9.3.1. We admit that the attrition rate of 15% is low, but our preliminary experience in 10 patients having undergone supervised training during immunochemotherapy demonstrated 100% adherence. As oncological exercise trials are subject to selection bias, study participants can be expected to be motivated to exercise. In addition, we will educate patients upon enrolment on the beneficial effects and safety of exercise during therapy.

#### 2.6.8 Data handling

Written consent for study participation, telemetric monitoring, and blood samples for the gynaecological bio database must be given before entering the trial. Employees involved in data collection are instructed and trained in all devices and measuring instruments to generate comparable measurements. Echocardiography will be performed by the blinded senior cardiologist. Data transmission (heart rates and electrocardiographic data) during training sessions follows national data policy and requires written patient consent prior to study inclusion.

The study nurse enters data into a password protected pseudonymized study database immediately after the examination. The decoding list of study IDs is stored separately from the database in paper and digital form and protected against unauthorized access.

The statistical evaluation, data clearance, assessment and interpretation are performed with the support of a professional statistician who receives data in pseudonymized form. Study results are only published based on anonymized data sets. The pseudonymization list is only available to the respective study nurse.

Personal information about potential and enrolled participants will only be collected, shared, and maintained by instructed study personnel. All patient information is encrypted and protected against unauthorized access by locking or password-protected systems. This applies before, during, and after the trial. After completion of the study, the pseudonymization list will be irrevocably deleted. At the request of the participants, the data on the pseudonymization list will be deleted at any time. Longer-term data storage only takes place in anonymized form to make the data available for future multicentre studies or meta-analyses. However, the data will be deleted 10 years after the study is discontinued or terminated. An interim analysis will be performed as soon as 30 patients have completed the t24 assessment. Expected VO2peak changes will be compared to measured changes to re-assess the sample size calculation. Decision upon modifications of sample size will be done by the blinded senior author.

#### 2.6.9 Statistical methods

For the analysis of the primary outcome (change in VO2peak from t0-t24), a linear regression model with change in VO2peak as the dependent variable and an indicator for the study group as well as the baseline VO2peak as independent variables will be fitted to the data. A two-sided Wald test is performed at a significance level of α = 5% and a corresponding 95% confidence interval is estimated for the mean group differences. Means, medians, standard deviations and ranges are presented for both groups. The primary analysis will follow the intention-to-treat (ITT) principle: All randomised participants will be included in the primary analysis of the group to which they were randomised. A multiple imputation approach will be used to replace missing data in the primary outcome variable. We will be using a “missing at random” strategy, with age-specific corrections for termination of exercise intervention due to side effects. An analysis of the per-protocol population will be performed as a sensitivity analysis. A difference of VO2peak of 2 ml/kg/min is deemed clinically relevant (6, 21, 22). To the best of our knowledge, no previous data on an exercise regimen comparable to our approach (5x/week, combination of balance, endurance and resistance training) exists. The threshold of 2 ml/kg/min was chosen, as large, dedicated exercise intervention trials have demonstrated comparable results (6, 23).

All secondary outcomes will be analysed with exploratory intent and will be descriptive in nature. Descriptive statistics will be generated for all relevant outcomes, stratified by study group. For quantitative secondary outcome variables, linear regression models will be fitted to the data, analogous to the primary outcome. For event- time outcomes (occurrence of CTRCD, ICI myocarditis, cardiovascular events, hospitalization due to decompensated heart failure, diagnosis of heart failure), Kaplan-Meier curves or cumulative incidence functions (considering competing risks) will be generated. Cox regression models, which include the study group as an independent variable, are fitted to the data. Event-specific hazard ratios with 95% confidence intervals are estimated and presented. Subgroup analyses are performed for important demographical and clinical baseline characteristics (e.g. age, body mass index, baseline VO2peak).

## 3 Discussion

Cardiorespiratory fitness is pivotal to reduce treatment-associated cardiovascular risk factors (24), mitigate the risk of premature mortality among BC survivors (22), and enhances the therapeutic effects of AC-based chemotherapy (25). However, maintaining adherence to exercise training is a major limiting factor to long-term stabilisation of exercise capacity in breast cancer patients (26). Thus, the implementation of remote training approaches (27, 28) in long-term cancer care is urgently needed. Although several studies have found the decline in VO2peak to be associated with reduced peripheral oxygen difference rather than limitations in stroke volume during cancer treatment (29, 30), insufficient information is available on the kinetics of the determinants of VO2peak during the cycles of chemotherapy.

The ExACT-ICI study includes all these aspects into one study. The combination of CPET and stress echocardiography during each visit provides objective data for the analysis of the complete oxygen cascade from mouth to the mitochondrion (31, 32) and for the assessment of physical training during combined immunochemotherapy in TNBC. Both acute (ICI-associated myocarditis, acute heart failure, acute coronary syndrome, hypertensive crisis) and chronic (arterial hypertension, coronary artery disease, chronic heart failure) events may only become manifest during exercise and require assessment by a cardiologist experienced in treating cancer patients. Standard cardiological care in oncological patients does not include exercise testing, which may lead to underdiagnosis of underlying disease. Our study will add to the literature by providing exercise data and the limitations of determinants (33–38) of VO2peak will be illustrated. Thus, our study may improve understanding of exercise pathophysiology during immunochemotherapy and may help to better define exercise regimens for these patients.

In addition, the supervised video-based training with telemedicine- monitoring enables patients in a vulnerable treatment phase to exercise at home, which is expected to increase adherence to training. By completion of this trial telemedicine-based training during chemotherapy should be implemented into general health offers for cancer patients to provide a broader access to ET in a cost-efficient way. The real-world implementation of cardio-oncological cooperation, as demonstrated by our trial, is recommended (19) but has not been put into practice in regular patient care.

The eligibility criteria of the ExACT-ICI study primarily address younger patients (18–65 years of age) without previous cardiovascular disease so that the handling of patient-triggered telemonitoring should not cause any problems with appropriate training in advance. Whether this approach can be transferred to older, multimorbid patients with metastatic disease is beyond the scope of this trial.

Like in other cancer exercise trials, selection bias will be present in this study, as only patients interested in ET will agree to participate. These patients might be disappointed when randomised into UC: We will educate UC on how to improve exercise capacity during treatment according to current guidelines (18, 19), although not with a tailored prescription as given in ET. Failure to educate UC on the beneficial effects of exercise during cancer treatment would be unethical. Although this may lead to a reduction in the group differences, it ensures standard-of- care treatment for all participating patients.

In summary, this is the first study to combine gynaecological, sports scientific and cardiological expertise to improve exercise capacity in TNBC patients receiving combined immunochemotherapy in a neoadjuvant setting using a step-up, non-linear periodization model including combined AET, RT and BT compared to UC. Due to the high methodological standards of this adequately powered trial, results can be expected to impact on upcoming guidelines of sports cardio-oncology.

## Supplementary material

Supplementary Figure 1 The duration of the preparation, initiation, recruitment, intervention and data analysis phases are displayed on a time axis.

Supplementary Figure 2 The workflow of screening, enrolment, randomization/allocation, follow-up and final analysis is provided.
