# Supplementary figures and images for: Exercise in regional breast cancer with neoadjuvant anthracycline-based chemotherapy with immune checkpoint-inhibition: study protocol for a prospective randomised controlled trial
Source: Front Oncol. 2026 Jun 5;16:1742136. doi: 10.3389/fonc.2026.1742136 (PMC13278924; doi:10.3389/fonc.2026.1742136)

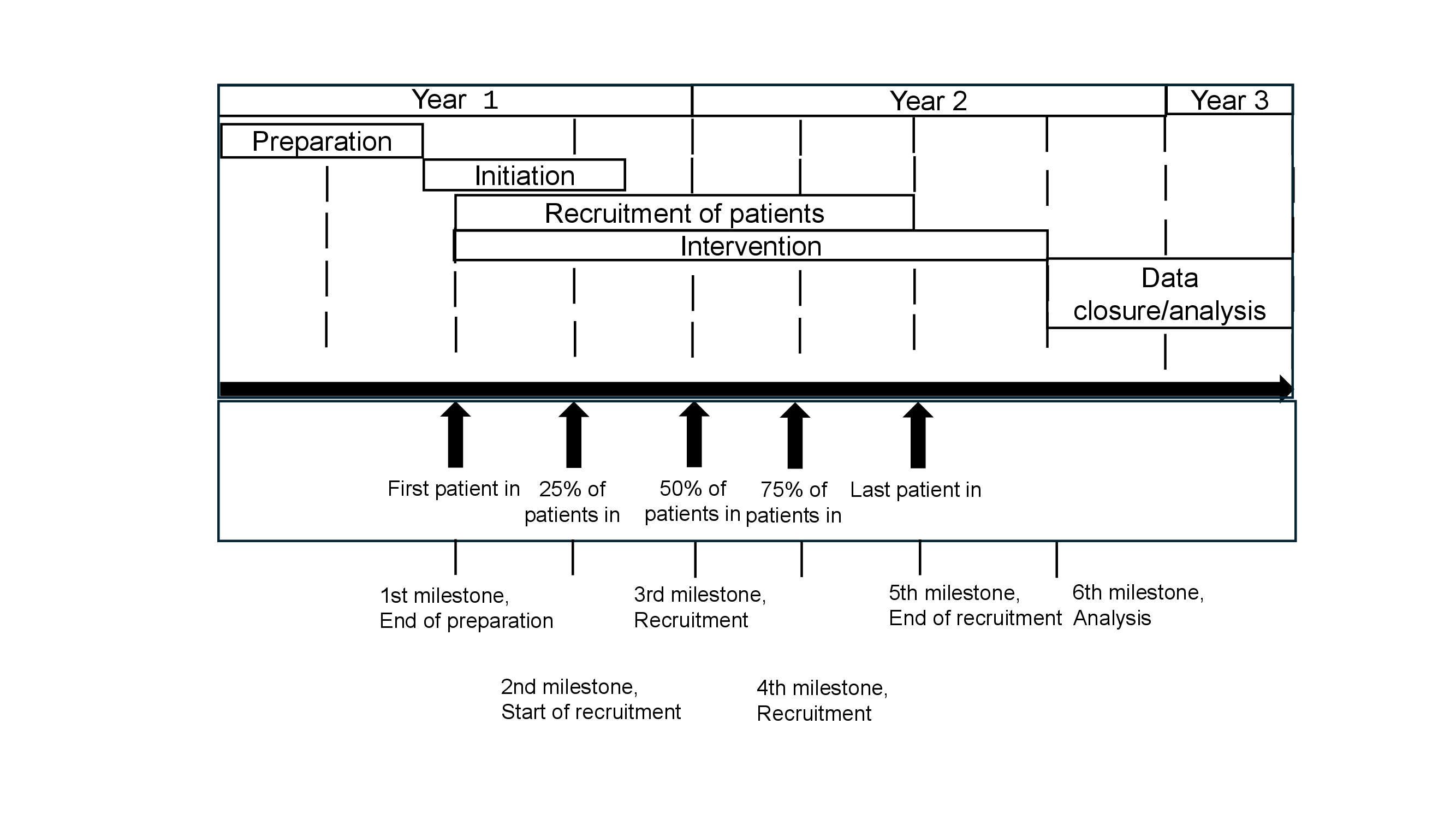

Supplement: Supplementary Figure 1 — The duration of the preparation, initiation, recruitment, intervention and data analysis phases are displayed on a time axis. [file Image1.tiff]
